# Changes of Health-Related Quality of Life Within the 1st Year After Stroke–Results From a Prospective Stroke Cohort Study

**DOI:** 10.3389/fneur.2021.715313

**Published:** 2021-10-04

**Authors:** Anabelle Kainz, Christa Meisinger, Jakob Linseisen, Inge Kirchberger, Philipp Zickler, Markus Naumann, Michael Ertl

**Affiliations:** ^1^Department of Neurology and Clinical Neurophysiology, University Hospital Augsburg, Augsburg, Germany; ^2^Chair of Epidemiology, University Augsburg, University Hospital Augsburg, Augsburg, Germany; ^3^Independent Research Group Clinical Epidemiology, Helmholtz Zentrum München, German Research Center for Environmental Health, Neuherberg, Germany

**Keywords:** stroke, disability, patient-reported outcome measures (PROMs), endovascular therapy (EVT), thrombolyic therapy

## Abstract

**Introduction:** As prospective data on long-term patient-reported outcome measures (PROMs) to assess Health related Quality of Life (HRQoL) after stroke are still scarce, this study examined the long-term course of PROMs and investigated influential factors such as recanalization therapies.

**Materials and Methods:** A total of 945 (mean age 69 years; 56% male) stroke patients were enrolled with a personal interview and chart review performed at index event. One hundred forty (15%) patients received thrombolysis (IVT) and 53 (5%) patients received endovascular therapy (ET) or both treatments as bridging therapy (BT). After 3 and 12 months, a follow-up was conducted using a postal questionnaire including subjective quality of life EQ-5D-5L (European Quality of Life 5 Dimensions). At all time-points, Modified Rankin Scale (mRS) was additionally used to quantify functional stroke severity. Differences between therapy groups were identified using *post-hoc*-tests. Linear and logistic regression analyses were used to identify predictors of outcomes.

**Results:** Recanalization therapies were associated with significant improvements of NIHSS (National Institutes of Health Stroke Scale [regression coefficient IVT 1.21 (*p* = 0.01) and ET/BT 7.6; *p* = 0.001] and mRS (modified Rankin Scale) [regression coefficient IVT 0.83; *p* = 0.001 and ET/BT 2.0; *p* = 0.001] between admission and discharge compared to patients with stroke unit therapy only, with a trend toward improvement of EQ-5D after 12 months [regression coefficient 4.67 (*p* = 0.17)] with IVT. HRQoL was considerably impaired by stroke and increased steadily in 3- and 12-months follow-up in patients with (mean EQ-5D from 56 to 68) and without recanalization therapy (mean EQ-5D from 62 to 68). In severe strokes a major and significant improvement was only detected during period of 3 to 12 months (*p* = 0.03 in patients with and *p* = 0.005 in patients without recanalization therapy).

**Conclusions:** Despite significant and continuous improvements after stroke the HRQoL after 12 months remained below the age-matched general population, but was still unexpectedly high in view of the accumulation of permanent disabilities in up to 30% of the patients. Especially in severe strokes, it is important to evaluate HRQoL beyond a 3-months follow-up as improvements became significant only between 3 months and 1 year.

## Introduction

Approximately 15 million people suffer a stroke every year. A high proportion of the cases is fatal, and one-third of stroke patients is affected by a serious, permanent disability (1). In Germany, stroke is the third-leading cause of death, with more than 60,000 fatalities out of ~260,000 cases (first-ever and recurrent strokes) each year (2). Nearly three-quarters of all strokes occur in people over the age of 65, with a decreasing chance of complete recovery or good functional outcome with increasing age (3). These numbers illustrate the importance of factors influencing patient outcomes, especially in the long run.

In the vast majority of stroke-related outcome studies, so-called clinician-reported outcome parameters like the modified Rankin Scale (mRS) quantify the patients' functional status. Although commonly used, this scale does not cover the patient's cognitive and social functions (4), nor essential domains such as symptom burden (e.g., fatigue) or emotional health (e.g., depression). Therefore, the mRS only provides limited information about the health status from the patient's perspective.

Patient-reported outcome measures (PROMs) specifically address the patient's view without interpretation by clinicians or anyone else (5). Health-related quality of life (HRQoL) can be assessed either with generic questionnaires, which can be applied to persons irrespective of a certain disorder, or with disease-specific measures, which are developed to capture the specific impairments associated with certain disease. Generic questionnaires allow comparisons across patients with different diseases and the general population. Moreover, generic questionnaires which are very short and easy to complete are available. This is an essential requirement when assessing HRQoL in the hospital setting after a severe acute event such as a stroke. The EuroQol Group 5-Dimension (EQ-5D) is a generic questionnaire which has already been applied and validated in patients with stroke (6–10). In this study we used EQ-5D-5L, which was introduced in 2005 as a new version of EQ-5D with increased reliability and sensitivity compared to EQ-5D-3L (11). It consists of two parts: the EQ-5D-5L descriptive system and a visual analog scale (EQ VAS). The descriptive system contains five questions about the severity in each of the five EQ-5D domains mobility, self-care, usual activities, pain/discomfort and anxiety/depression which can be combined into a single utility value (EQ-5D index). The EQ VAS provides an overall assessment of the current general health state. Completing the questionnaire does not take longer than 3–5 min in average. Information on PROMs allow clinicians to improve shared decision-making and provide individualized care to improve the patient's health-related quality of life (HRQoL) (4).

Intravenous thrombolysis therapy (IVT) and the recent implementation of endovascular thrombectomy (ET) as well as their combination termed “bridging therapy” (BT) led to a significant improvement in the short-term functional outcome of these severely affected subgroups of ischemic strokes with large vessel occlusions of the anterior circulation (12–14).

Yet, prospective data regarding the long-term impact on PROMs are almost entirely lacking in this patient population.

Therefore, we focused on the analysis of PROMs and disabilities in the long-term outcome of stroke patients in a large prospective cohort study with and without acute recanalization therapies.

## Materials and Methods

### Sample Size Estimation

Every year, ~1,700 patients with acute strokes are treated at the University Hospital Augsburg. It was estimated that about 60–70% of the patients would take part in the study (15). Thus, we assumed that about 900–1,000 patients could be recruited within the study period of 1 year. With an estimated effect estimate (HR) of 1.7 for the covariate of interest, a variance of 0.36 and a rho^2^ = 0.3, the inclusion of 997 patients would be sufficient to detect a significant difference with a statistical power of 80% at a significance level of 5%.

### Study Population, Data Collection and Follow-Up

**Figure 1:**
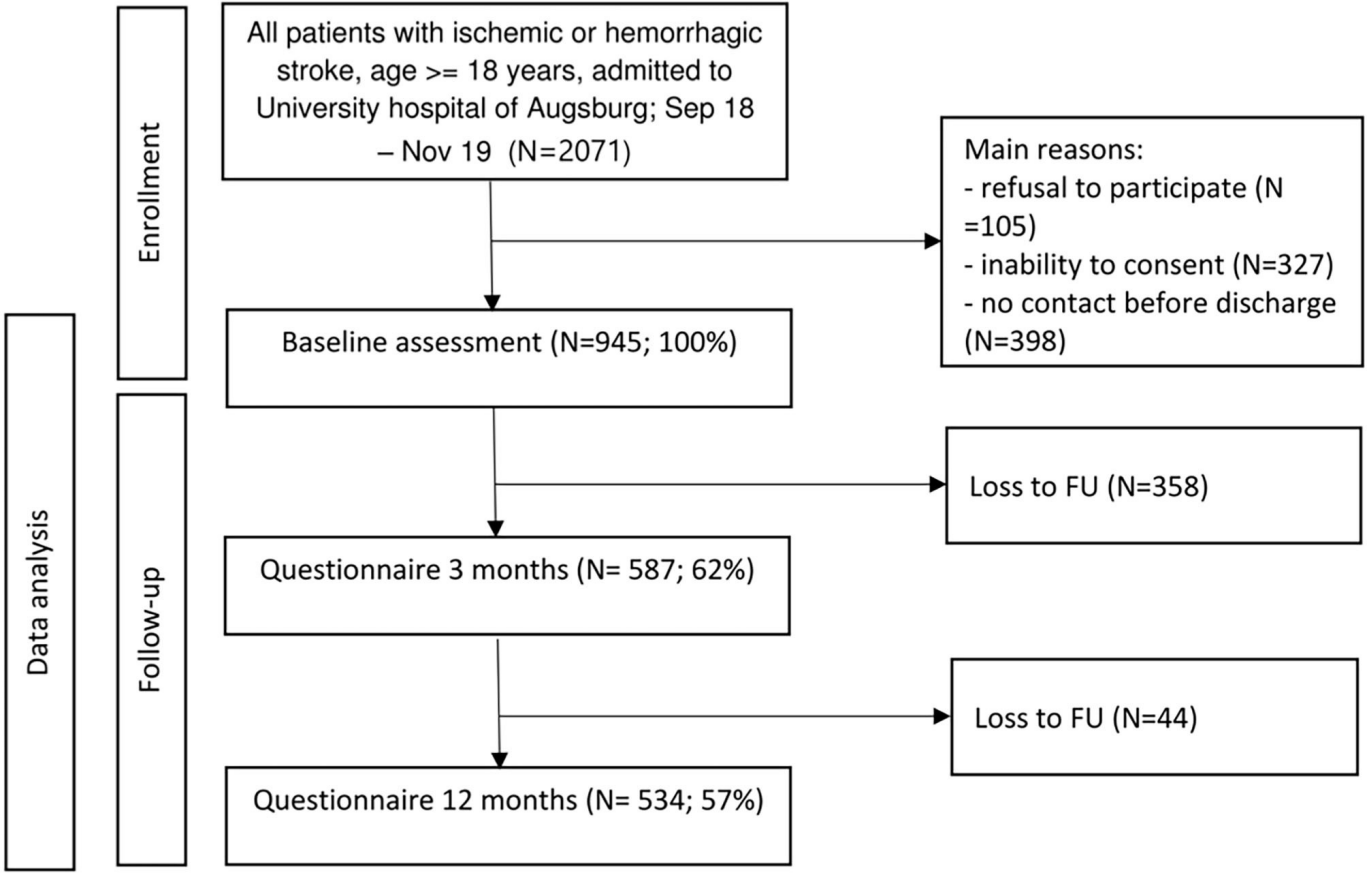
Consort chart of patient enrollment and follow-up.

Between September 2018 and November 2019, all consecutive adult patients (18 years and older) with an incident as well as recurrent ischemic or hemorrhagic stroke, who were admitted to the University Hospital of Augsburg, were screened for enrollment. Proportions of baseline and follow-up assessment are shown in Figure 1. Detailed information about methods for recruitment, conduction of patient interviews and obtainment of follow-up data has been published elsewhere (15).

In summary, trained study nurses prospectively recorded all stroke cases and interviewed patients or legal caregivers after written informed consent. In the interview, demographic information, symptoms upon presentation, diagnosis, lifestyle factors and comorbidities (e.g., carcinoma, cardiac comorbidity, diabetes mellitus) were gathered. Clinical data on comorbidities, risk factors, medication prescribed before hospital stay and at discharge, diagnostic procedures, clinical characteristics, disabilities, laboratory parameters, and treatment regimens during the hospital stay were additionally registered by a chart review.

Three and 12 months after hospital discharge, all study participants received postal questionnaires or telephone interviews with questions on disease symptoms, disabilities, physical activity (German-PAQ-50+) (16), health-related quality of life measured with EQ-5D-5L (European Quality of Life 5 Dimensions) (7, 17) recurrent events and healthcare utilization.

### Outcomes

During inpatient stay, clinical outcomes were measured using the National Institute of Health Stroke Scale (NIHSS: score range 0–42) and mRS [score range 0 (no functional deficits) to 6 (deceased)] with higher scores indicating a greater neurological deficit or a higher degree of physical disability (18, 19). NIHSS and mRS were recorded from the patient chart at the timepoints of hospital admission and discharge.

Patient-reported health status was assessed by a visual analog scale (EQ VAS), which evaluates the patient's current overall subjective health from 0 (worst imaginable health) to 100 (best possible health). EQ-5D was assessed in the subacute stroke phase at the end of inpatient stay.

Reliability and validity of the main measures NIHSS (18, 20), mRS (19, 20) and EQ-5D (6–10) used in this study are confirmed in the literature, both in its original and German version: In detail, interrater reliability of NIHSS [mean κ (kappa) = 0.80 in German version (20) and κ = 0.95 in original version (18)] and mRS [mean κ = 0.76 in German version (20) and κ = 0.78 in original version (19)] is very high, as well as the intraclass correlations of EQ-5D ranging from 0.67 to 0.8 (8).

Additionally, information on care dependency (e.g., eligibility of a disabled person's card) and use of medical aids (e.g., walking stick or wheelchair) was collected.

In the follow-up stage, mRS as a functional outcome and EQ-5D assessing the patients' subjective health status, were both evaluated by a postal questionnaire sent to the patients after 3 and 12 months.

### Statistical Analysis

Baseline characteristics are presented as mean ± standard deviation (SD) for normally distributed or median ± interquartile range (IQR) for non-normally distributed continuous variables and percentage for categorical variables (Table 1). In a first step, Chi^2^-Tests were performed for categorical variables if each cell contained at least 5 expected values. If this was not the case, a Fisher's exact test was calculated. Continuous variables were tested for normal distribution using Shapiro-Wilk-Test and Q-Q plots. Non-parametric Mann-Whitney-U-Tests for non-normally distributed variables and *t*-tests for independent samples for normally distributed variables were used to compare patients with and without stroke-specific acute therapy. In a second step, differences between the four treatment groups (IVT, IAT, BT, and no treatment) were examined using Chi^2^ or Fisher's exact test for categorical variables and non-parametric Kruskal-Wallis-Tests for continuous variables. If these were significant, treatment groups were compared pairwise using Bonferroni adjusted *post-hoc*-tests.

**Table 1:** Baseline characteristics of stroke patients with and without recanalization therapy [thrombolysis (IVT), endovascular therapy (ET) or bridging therapy (BT)].

**Variables (number of valid cases)**	**Recanalization therapy (IVT and/or ET)**	**Stroke unit therapy only**	*p*-value^*^
Number of patients (945)	193 (20)	752 (80)	
Interviews and file processing (787)–no. (%)	158 (82)	629 (84)	
File processing only (158)–no. (%)	35 (18)	123 (16)	
**Characteristics**			
**Age (945)–mean (SD) by years**	71 (12)	69 (13)	0.09^d^
•> 80 years - no. (%)	50 (26)	184 (25)	
•50–79 years - no. (%)	136 (70)	508 (67)	
• <50 years - no. (%)	7 (4)	60 (8)	
**Sex (945)–no. (%)**			0.06^a^
•Female	96 (50)	318 (42)	
•Male	97 (50)	434 (58)	
BMI (932)–mean (SD)	27 (5)	27 (5)	0.55^d^
**Risk factors**			
Nicotine abuse (945)–no. (%)	88 (46)	381 (51)	0.48^a^
Hypertension (945)–no. (%)	163 (85)	601 (80)	0.15^a^
Atrial fibrillation (945)–no. (%)	47 (24)	141 (19)	0.08^a^
Hyperlipidemia (920)–no. (%)	91 (48)	356 (49)	0.83^a^
Diabetes mellitus (936)–no. (%)	40 (21)	169 (23)	0.58^a^
**Etiology**			
**Stroke type** **(945)–no. (%)**			**0.002^a^**
•Ischemic	193 (100)	715 (95)	
•Hemorrhagic	0 (0)^**^	37 (5)^**^	
**Stroke etiology** **(873)–no. (%)**			**0.01^b^**
•Macroangiopathic	47 (25)	176 (26)	
•Microangiopathic	31 (16)	132 (19)	
•Cardio-embolic	66 (35)^**^	153 (22)^**^	
•Others	3 (1)	18 (3)	
•Unknown	43 (23)^**^	204 (30)^**^	
**Severity**			
NIHSS admission (892)–median (IQR)	4 (7)	1 (3)	**0.001^c^**
NIHSS discharge (834)–median (IQR)	1 (2)	0 (1)	**0.001^c^**
**mRS admission (895)–no. (%)**			**0.001^b^**
•0	1 (1)^**^	139 (20)^**^	
•1	10 (5)^**^	144 (21)^**^	
•2	17 (9)^**^	180 (26)^**^	
•3	52 (27)^**^	128 (18)^**^	
•4	78 (40)^**^	94 (13)^**^	
•5	35 (18)^**^	17 (2)^**^	
**mRS discharge** **(894)–no. (%)**			**0.001^b^**
•0	305 (43)^**^	41 (21)^**^	
•1	164 (23)	48 (25)	
•2	117 (17)	33 (17)	
•3	64 (9)^**^	38 (20)^**^	
•4	45 (6)	20 (10)	
•5	3 (1)^**^	9 (5)^**^	
•6	4 (1)	3 (2)	
EQ VAS during hospitalization (752)–mean (SD)	56 (25)	62 (21)	**0.03^d^**

a *Chi2-test;*

b *Fisher's exact test;*

c *Mann-Whitney-U-Test;*

d *t-test for independent samples*.

* *p-value indicates differences in baseline variables between recanalization therapy (IVT, ET, BT) and stroke unit therapy only*.

** *indicates significant differences between recanalization therapy and stroke unit therapy only of categorical variables*.

*Significant p-values were highlighted in bold*.

Patients with hemorrhagic stroke (*n* = 37; 5%) were excluded from statistical analyses on stroke severity and follow-up as well as in regression analyses.

For the identification of the outcome both from clinical and patient's perspective, linear regression models were fitted for improvement of NIHSS and mRS between admission and discharge as well as EQ VAS during hospitalization, after 3 and 12 months. Ordinal logistic regressions were fitted for subjective functional limitations after 3 and 12 months. Potential confounders were defined as variables found in the literature to be related to the outcome and associated with the exposure but not intermediate variables in the causal pathway between exposure and outcome. All regression models were adjusted for age, sex, highest school-leaving qualification, smoking, alcohol abuse (with AUDIT-C), physical activity [with IPAQ (International physical activity questionnaire)] and the presence of at least one comorbidity. The treatment groups IVT and IAT/BT were compared to patients without acute therapy. All model assumptions were fulfilled.

A subgroup analysis including severely affected patients (mRS 3-5) was performed using non-parametric Wilcoxon-Tests to identify relevant changes in patient-reported health between follow-up timepoints.

## Results

### Study Population at Baseline

During the study period, 945 consecutive patients were included. Baseline characteristics are shown in Table 1. Patients were divided into two groups: patients without recanalization therapy (80%) and patients having received a stroke-specific acute therapy (15% IVT and 5% ET or BT).

In most of the baseline characteristics, there were no significant differences between both patient groups. Still, the following significant difference in stroke etiology and severity was mentionable: In patients with recanalization therapy, cardiogenic causes were significantly more frequent compared to patients with stroke unit therapy only. Patients with stroke-specific acute therapy had significantly higher NIHSS and mRS values at admission and discharge and significantly lower EQ VAS values.

### Association Between Acute Recanalization Therapies and Outcomes of Stroke Patients

#### During Inpatient Stay

The influence of stroke-specific acute therapies on clinical and patient-reported outcome measures is shown in Table 2. Recanalization therapies were associated with a significantly larger clinical improvement in terms of NIHSS and mRS between admission and discharge compared to patients without recanalization therapy. At the time point of discharge, the treatment group had the identical mean NIHSS compared to the no-recanalization group at admission. Nevertheless, looking at overall health status from the patient's perspective, patients with stroke-specific acute therapy reported significantly lower EQ VAS scores than the group of patients without recanalization therapy during hospitalization (*p* = 0.03, see Table 1). After adjusting for confounding variables, this effect was no longer significant.

**Table 2:** Association between stroke-specific acute therapies and patient outcome.

Outcome^*^	**Effect estimate**	**Adjusted estimate (95% CI)**	***p*-value**
**NIHSS improvement between admission and discharge**			
•IVT	Regression coefficient β	1.21 (0.22 to 2.20)	**0.01^a^**
•ET or BT	Regression coefficient β	7.6 (6.14 to 9.11)	**0.001^a^**
**mRS improvement between admission and discharge**			
•IVT	Regression coefficient β	0.83 (0.42 to 1.23)	**0.001^a^**
•ET or BT	Regression coefficient β	2.0 (1.37 to 2.58)	**0.001^a^**
**EQ VAS during hospitalization**			
•IVT	Regression coefficient β	−5.17 (−12.40 to 2.05)	0.16^a^
•ET or BT	Regression coefficient β	−2.86 (−13.72 to 8.00)	0.60^a^
**EQ VAS after 3 months**			
•IVT	Regression coefficient β	−1.96 (−8.18 to 4.25)	0.53^a^
•ET or BT	Regression coefficient β	−1.41 (−10.77 to 7.93)	0.76^a^
**Subjective functional limitations after 3 months**			
•IVT	Odds ratio	1.43 (0.89 to 2.29)	0.13^b^
•ET or BT	Odds ratio	1.69 (0.84 to 3.44)	0.14^b^
**EQ VAS 12 months**			
•IVT	Regression coefficient β	4.67 (−2.03 to 11.37)	0.17^a^
•ET or BT	Regression coefficient β	−2.68 (-12.75 to 7.40)	0.60^a^
**Subjective functional limitations after 12 months**			
•IVT	Odds ratio	1.00 (0.56 to 1.76)	0.98^b^
•ET or combined	Odds ratio	1.75 (0.74 to 4.16)	0.20^b^

a *linear regression;*

b *ordinal logistic regression*.

* *Patients with IVT and ET/BT therapy were compared to patients without recanalization therapy*.

*Significant p-values were highlighted in bold*.

#### Follow Up at 3 and 12 Months

A total of 587 (62%) patients took part in the 3-month follow-up and 534 (57%) in the 12-months-follow-up. The response rate of the two groups with and without stroke-specific acute therapy was similar. The results of the follow-up assessments are shown in Table 3.

**Table 3:** Follow-up after 3 and 12 months of stroke patients with and without recanalization therapy [thrombolysis (IVT), endovascular therapy (ET) or bridging therapy (BT)].

**Variables (number of valid cases)**	**Recanalization therapy**	**Stroke unit therapy only**
3-month-follow-up (587) – no. (%)	113 (59)	474 (63)
12-month-follow-up (534) – no. (%)	110 (57)	424 (56)
**Subjective health status**		
EQ VAS during hospitalization (752) – mean (SD)	56 (25)	62 (21)
EQ VAS at 3 months (550) – mean (SD)	65 (22)	67 (19)
EQ VAS change baseline – 3 months (527) – mean (SD)	8 (20)	4 (21)
EQ VAS at 12 months (500) – mean (SD)	68 (19)	68 (19)
EQ VAS change baseline – 12 months (485) – mean (SD)	5 (21)	5 (22)
**Objective functional limitations**		
Disability card at 3 months (554) – no (%)	24 (21)	113 (26)
Need of care at 3 months (546) – no (%)	21 (19)	66 (15)
Medical aids (eg. wheelchair) at 3 months (496) – no (%)	36 (33)	99 (26)
Disability card at 12 months (494) – no (%)	30 (29)	114 (29)
Need of care at 12 months (496) – no (%)	21 (19)	58 (15)
Medical aids (e.g., wheelchair) at 12 months (441) – no (%)	25 (25)	89 (26)

**Figure 2:**
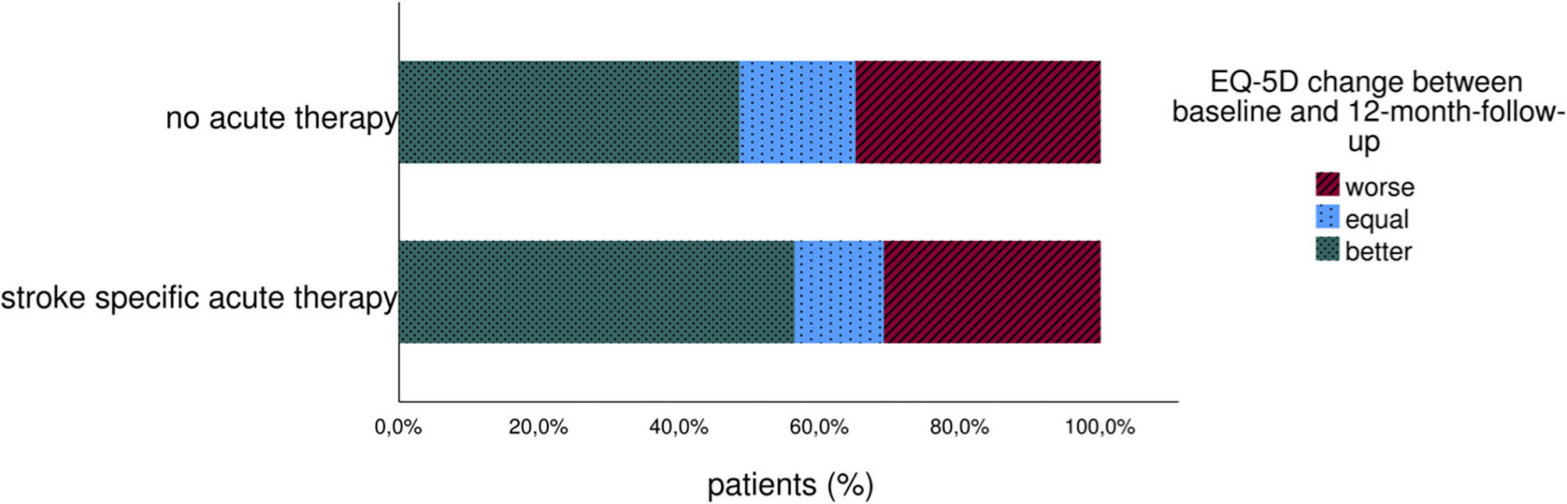
Change of long-term patient-reported overall health (EQ VAS) of stroke patients with and without acute therapy.

Looking at EQ VAS change between baseline and 3 months follow-up, the patient's health status increased in both groups, with no significant changes between therapy or stroke unit therapy only. After 12 months, EQ VAS of treated patients was equal to the well-being of patients without recanalization therapy (mean EQ VAS 68 ± 19 in both groups). Altogether, 56% of patients with and 48% of patients without recanalization therapy reported increased overall health between the index event and 12 months on EQ VAS, as visualized in Figure 2.

### Subgroup Analysis of Patients With High-Grade Functional Deficits

Because of the relatively low average mRS in the entire cohort, we added a subgroup analysis of patients with relevant functional deficits (mRS 3-5, dependency on assistance in everyday life).

**Figure 3:**
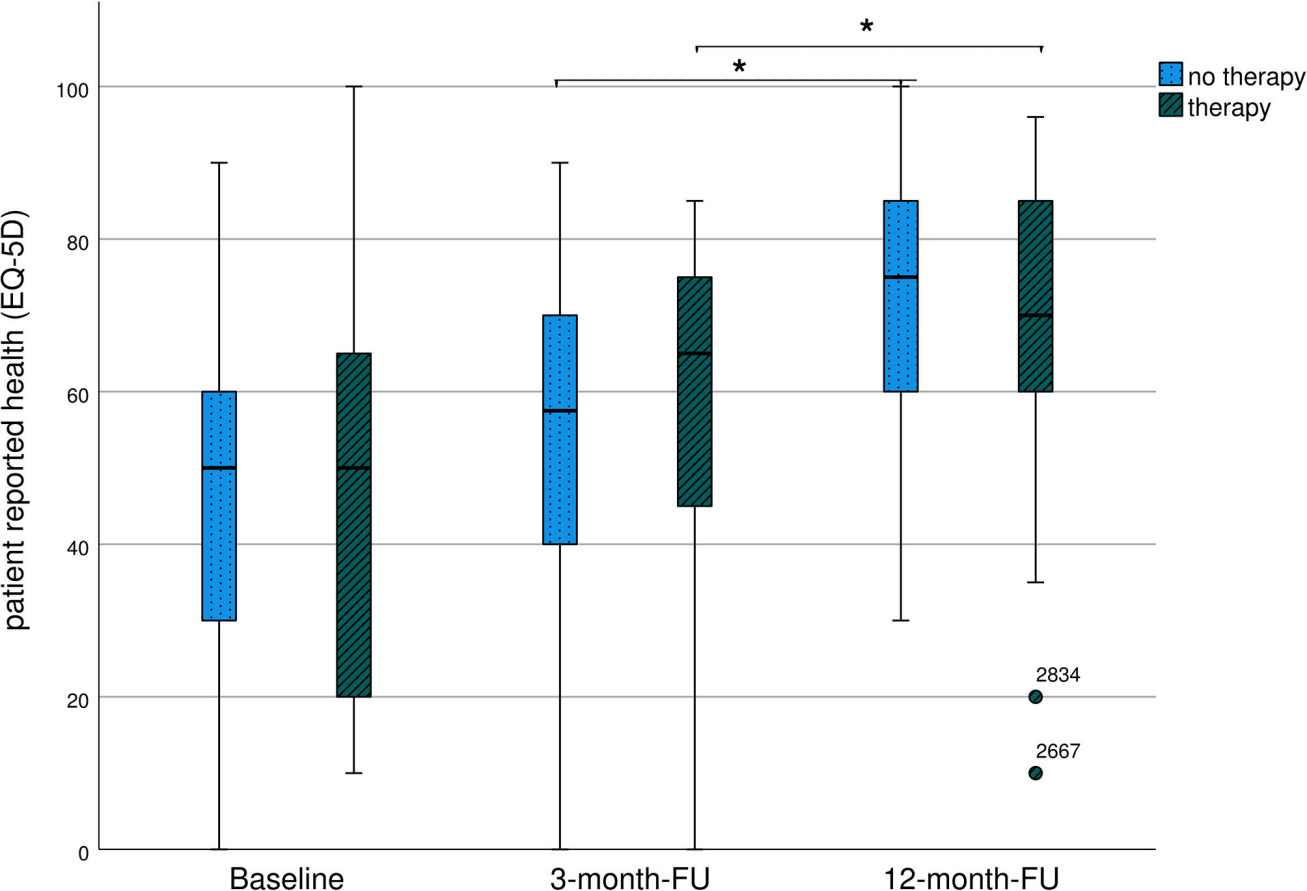
Long-term course of patient-reported health (EQ VAS) of severely affected patients (mRS 3-5)-representation as boxplots. *Significant differences between groups.

The results are presented in Table 4; Figure 3. EQ VAS steadily increased during the follow-up period, with a significant improvement between 3 and 12 months.

**Table 4:** Long-term course of patient-reported health (EQ VAS) of severely affected patients (mRS 3-5).

		**Baseline**	**3-month-FU**	*p*-value^*^	**12-month-FU**	***p*-value^+^**
EQ VAS – mean (SD)	Acute therapy (*n* = 67)	47 (25)	54 (26)	0.44	68 (20)	**0.03**
	No acute therapy (*n* = 112)	47 (21)	52 (21)	0.14	72 (15)	**0.005**

* *p-value of EQ VAS between baseline and 3 month follow-up*.

+ *p-value of EQ VAS between 3 and 12 month follow-up*.

*Significant p-values were highlighted in bold*.

### Other Parameters With Effect on Patient-Related Outcomes

Independent from the effect of therapy, patients with higher age (β = −0.17; 95% CI: −0.32, −0.02; *p* = 0.02), NIHSS on admission (β = −0.86; 95% CI: −1.45, −0.27; *p* = 0.004) and mRS at discharge (β = −1.82; 95% CI −3.3, −0.34; *p* = 0.02) reported significantly lower EQ VAS scores after 3 months. After 12 months a history of at least one previous stroke (β = −6.30; 95% CI: −11.52, −1.08; *p* = 0.02) was associated with significantly worse patient's health. Stroke etiology had no significant effect on the patient's health well-being.

### Objective Functional Limitations

Three months after stroke, a high percentage of patients (20–30%) were permanently dependent on care, had a severely disabled pass or were dependent on medical aids such as walking sticks or wheelchairs. After 12 months of recovery from stroke, the numbers remained high, as shown in Table 3. Since, at baseline, only six patients (0.8%) lived in assisted living and not a single patient lived in a nursing home, these limitations were most likely caused by stroke.

## Discussion

This is one of the first prospective cohort studies that has analyzed long-term outcomes in stroke patients with focus on HRQoL and effects of acute recanalization stroke therapies. We could show, that HRQoL continuously improved in all patient groups. However, the most pronounced effect occurred between 3 and 12 months after stroke, especially in patients with relevant functional deficits (mRS 3-5). This observation was valid independent from stroke-specific acute therapies.

Acute recanalization therapies have a positive effect on functional deficits after stroke (21, 22). In the present study, these highly effective therapies strongly impacted short-term functional deficits, with a significant improvement of NIHSS and mRS values in patients with recanalization therapy. In average, this resulted in identical NIHSS values comparing treated patients at the timepoint of discharge and only mildly affected patients with no need of therapy at the time of admission (NIHSS = 1). With respect to HRQoL, patients in the therapy group had a significantly lower EQ VAS compared to the stroke patients without acute therapy, but after adjustment for confounding variables, patients having profited from recanalization therapies did not have a significantly different EQ VAS compared to the patients with stroke unit therapy only, neither in the subacute phase nor in the follow-up period. At first glance, this was rather surprising, as another study could detect a prominent treatment effect, especially for endovascular therapy (23). As functional deficits and HRQoL correlate in stroke patients (24), the strong functional improvement during hospitalization in our treated patients might explain the low treatment effect in the statistical analysis of the present cohort. The EQ-5D questionnaire was assessed at the end of inpatient stay when functional deficits of treated patients were already ameliorated to a level comparable to the only mildly affected patients in the no recanalization therapy group. Another aspect might be that specific treatments might only be one amongst many other factors influencing patient-centered outcomes. Observational studies in patients with malignant diseases, e.g., bladder or breast cancer, revealed a similar effect: age, comorbidities or psychological distress had a greater influence on EQ-5D than the specific treatments received (25, 26). For HRQoL individual coping strategies have a significant impact on the patient's subjective well-being. Active coping strategies have a beneficial effect on HRQoL after stroke with positive aspects being social support, extrovertive personality traits and active information seeking (27).

For interpretation of EQ-5D values in stroke patients, it is necessary to compare these patients to healthy persons at the same age and to patients with comparable diseases. Patients in the present cohort rated their overall health status worse than a representative sample of the age-matched German population [mean EQ VAS = 75 in persons 60–79 years of age (28)]. Interestingly, even mildly affected patients without recanalization treatment (mean NIHSS 2) rated their subjective health (EQ 62 ± 21) as low as patients with serious diseases such as advanced colorectal cancer [mean EQ VAS 62 (29)] or heart disease [mean EQ VAS 61 (28)]. Over time, HRQoL continued to improve in all patient groups, with the most significant effect between 3 and 12 months, especially in the subgroup of patients with severe functional deficits (mRS 3-5). The EQ VAS level improved to a value of 68 after 12 months, but did not reach the general population's comparative value [mean EQ VAS = 75 (28)]. Our results are in line with other studies, which found that even after 5 years HRQoL of stroke survivors remained below the general population level (30).

Altogether, the present results suggest that a relatively short follow-up time of 3 months - as recommended in a recent meta-analysis (22) and practiced in the majority of controlled trials - may not be sufficient to predict the patients' long-term HRQoL. Our data show that this applies for the initially more severely affected patients of the therapy groups and overall especially for the subgroup with severe clinical deficits (mRS 3-5) that needed up to 12 months to recover and improve their HRQoL. For future evaluation an extended follow-up period of 24 months is already planned, as we need to increase our understanding of long-term functional and mental recovery in stroke patients. HRQoL may continue to improve in the 2nd year, which is also seen in a study of young stroke patients (31). As the current literature suggest a poor long-term HRQoL of stroke survivors (32–34), this information may be of great value especially for patients with relevant functional deficits. One explanation for delayed functional recovery, especially in more severely affected patients, might be the process of neuronal remodeling, which is restricted in patients with extended neuronal damage and can be predicted by particular imaging methods (35). Going along with the observation of delayed recovery was that the need for medical aids (e.g., wheelchair) improved from 33% at 3 months to 25% at 12 months in the therapy group while it remained at 26% for both time points in the group without recanalization therapy. Increased mobility might thus be an important factor, whereas the need of care remained unchanged for both time points at 19 and 15% in the recanalization vs. non-recanalization therapy group, respectively.

Individual prognosis is essential for patients in the subacute period after stroke. In our data, younger age and better clinical outcome measures (NIHSS and mRS) positively influenced EQ VAS after 3 months. The chance for a good EQ VAS after 12 months was lower in patients with recurrent strokes. In the current literature, factors such as male sex, white race, social support and active coping strategies have also been identified as positive predictors for better HRQoL, whereas stroke severity and functional impairment were associated with a more unsatisfactory outcome in stroke survivors (36, 37).

As PROMs reflect the patient-immanent strategies to cope with a disease, they do not necessarily correlate with objective outcome parameters such as need for professional care or medical aids. Interestingly, we observed a discrepancy between a high level of subjective health quality despite a significant proportion of our patients being in permanent need of care 12 months after the index event. These limitations based on objective criteria should be scrutinized in future observational outcome studies, as this information is especially relevant from a health care point of view a high economic burden for the health care system (38, 39). Standardized registration of PROMs in the acute and follow-up phase could improve self-management in patients and support physicians to gain information about long-term HRQoL in their patients. To support this, digital measurement systems have already been tested but further research in this area is still needed (40).

As a result of our study and the current literature, PROMs are a valuable supplement, but no substitute to clinician-reported outcome measures. Despite a considerable proportion of objective impairments, the subjective health perception of stroke patients after 1 year is surprisingly high (mean EQ VAS 68 ± 19) and well above that of other chronic diseases such as cancer [mean EQ VAS 62 (29)] or heart disease [mean EQ VAS 61 (28)]. This information is highly relevant for clinicians when counseling stroke patients and their relatives.

To guide individualized care concerning PROMs, clinicians need to know, which patients are at particular risk of long-term impairment of individual HRQoL. So far, we know that certain comorbidities as depression (41) and other factors as unemployment and lack of family support (42) negatively affect these outcomes. Therefore, physicians should focus on early interventions of e.g. post stroke depression in the (sub)acute phase. During rehabilitation and afterwards measures as reintegration in the job market or implementation of stroke support groups have a positive effect, as documented in the literature (43). Other aspects as the level of education and migrational background still need to be investigated in future studies.

### Strengths and Limitations

The long follow-up period of prospectively analyzed stroke patients with focus on PROMs combined with the detailed information on acute stroke treatment and objective functional limitations represents a major strength of our study.

The use of the EQ-5D enables a comparison with other diseases. However, the EQ-5D does not allow a detailed description of specific domains of HRQoL such as social, cognitive and emotional impairments. Thus, the results of this study should be confirmed using a more stroke-specific questionnaire. However, 47% of the consecutive stroke patients could not be included in the study e.g., due to refusal (5%) or inability (16%) to participate and 38% of the baseline assed patients were lost to follow-up. This may produce bias because more severely affected stroke patients may have been more likely to refuse participation. The patients in the different treatment groups are not matched and cannot be compared directly. This aspect was addressed by a subgroup analysis of severely affected patients (mRS 3-5) and by adjustment of confounders in regression models.

Nevertheless, 57% of the patients assessed at baseline (*n* = 534) still participated after 12 months. Despite comparable recanalization therapy rates between our cohort and the Bavarian average (44), the number of patients with compared to patients without recanalization therapy was relatively small making it difficult for smaller effects to become significant.

## Conclusion

In conclusion, this study provides novel insights into long-term patient-reported outcomes and objective functional impairments after stroke. The study demonstrated that initial functional deficits, age and recurrent strokes predict HRQoL, whereas acute therapies beyond their immediate effect were not clearly associated with patient-reported outcomes. The subjective health related quality of life steadily increased for all patients and recovered to high levels at 12 months despite a high proportion of persisting disability in up to 29% of the patients. Especially severely affected patients needed longer periods of time and improved most between 3 and 12 months. Nevertheless, after 12 months, the overall health status from the patient's perspective did not regain the level of the age-matched general population. Since objective outcome parameters do not necessarily match to the same degree as patient-related outcome measures, these aspects are important for adequate patient-centered counseling and should be included in future observational studies.

## Data Availability Statement

The raw data supporting the conclusions of this article will be made available by the authors, without undue reservation.

## Ethics Statement

The studies involving human participants were reviewed and approved by Ludwig-Maximilians University Munich. The patients/participants provided their written informed consent to participate in this study.

## Author Contributions

Formal analysis was done by CM and AK. The manuscript was drafted by AK and ME. All authors contributed to the study conception and design, commented on previous versions of the manuscript, read and approved the final manuscript.

## Funding

The prospective stroke cohort Augsburg (SCHANA) was financially supported by the Faculty of Medicine, University of Augsburg, Germany.

## Conflict of Interest

The authors declare that the research was conducted in the absence of any commercial or financial relationships that could be construed as a potential conflict of interest.

## Publisher's Note

All claims expressed in this article are solely those of the authors and do not necessarily represent those of their affiliated organizations, or those of the publisher, the editors and the reviewers. Any product that may be evaluated in this article, or claim that may be made by its manufacturer, is not guaranteed or endorsed by the publisher.

## Abbreviations

NIHSS: National Institutes of Health Stroke Scale
mRS: modified Ranking Scale
PROMs: patient-reported outcome measures
HRQoL: health-related quality of life
EQ VAS: European Quality of Life 5 Dimensions visual analogue scale
IVT: intravenous thrombolysis
ET: endovascular therapy
BT: bridging therapy.

## References

[1] Benjamin EJ Blaha MJ Chiuve SE Cushman M Das SR Deo R. Heart disease and stroke statistics-2017 update: a report from the American heart association. Circulation. (2017) 135:e146–603. 10.1161/CIR.0000000000000491 28122885 PMC5408160

[2] Heuschmann PU Biegler MK Busse O Elsner S Grau A Hasenbein U. Development and implementation of evidence-based indicators for measuring quality of acute stroke care: the Quality Indicator Board of the German Stroke Registers Study Group (ADSR). Stroke. (2006) 37:2573–8. 10.1161/01.STR.0000241086.92084.c0 16960092

[3] Donnan GA Fisher M Macleod M Davis SM. Stroke. Lancet. (2008) 371:1612–23. 10.1016/S0140-6736(08)60694-7 18468545

[4] Reeves M Lisabeth L Williams L Katzan I Kapral M Deutsch A. Patient-reported outcome measures (PROMs) for acute stroke: rationale, methods and future directions. Stroke. (2018) 49:1549–56. 10.1161/STROKEAHA.117.018912 29789396

[5] Price-Haywood EG Harden-Barrios J Carr C Reddy L Bazzano LA van Driel ML. Patient-reported outcomes in stroke clinical trials 2002-2016: a systematic review. Qual Life Res. (2019) 28:1119–28. 10.1007/s11136-018-2053-7 30465318

[6] Dorman PJ Waddell F Slattery J Dennis M Sandercock P. Is the EuroQol a valid measure of health-related quality of life after stroke? Stroke. (1997) 28:1876–82. 10.1161/01.STR.28.10.1876 9341688

[7] Golicki D Niewada M Buczek J Karlińska A Kobayashi A Janssen MF. Validity of EQ-5D-5L in stroke. Qual Life Res. (2015) 24:845–50. 10.1007/s11136-014-0834-1 25347978 PMC4366565

[8] Hunger M Sabariego C Stollenwerk B Cieza A Leidl R. Validity, reliability and responsiveness of the EQ-5D in German stroke patients undergoing rehabilitation. Qual Life Res. (2012) 21:1205–16. 10.1007/s11136-011-0024-3 21971874

[9] Pinto EB Maso I Vilela RNR Santos LC Oliveira-Filho J. Validation of the EuroQol quality of life questionnaire on stroke victims. Arq Neuropsiquiatr. (2011) 69:320–3. 10.1590/S0004-282X2011000300010 21625758

[10] Quinn TJ Dawson J Walters MR Lees KR. Functional outcome measures in contemporary stroke trials. Int J Stroke. (2009) 4:200–5. 10.1111/j.1747-4949.2009.00271.x 19659822

[11] EQ-5D-5L-English-User-Guide_version-3.0-Sept-2019-secured.

[12] Campbell BCV Donnan GA Lees KR Hacke W Khatri P Hill MD. Endovascular stent thrombectomy: the new standard of care for large vessel ischaemic stroke. Lancet Neurol. (2015) 14:846–54. 10.1016/S1474-4422(15)00140-4 26119323

[13] Mazighi M Meseguer E Labreuche J Amarenco P. Bridging therapy in acute ischemic stroke: a systematic review and meta-analysis. Stroke. (2012) 43:1302–8. 10.1161/STROKEAHA.111.635029 22529310

[14] Shi Z-S Loh Y Walker G Duckwiler GR. Endovascular thrombectomy for acute ischemic stroke in failed intravenous tissue plasminogen activator versus non-intravenous tissue plasminogen activator patients: revascularization and outcomes stratified by the site of arterial occlusions. Stroke. (2010) 41:1185–92. 10.1161/STROKEAHA.109.568451 20431084

[15] Ertl M Meisinger C Linseisen J Baumeister S-E Zickler P Naumann M. Long-term outcomes in patients with stroke after in-hospital treatment-study protocol of the prospective stroke cohort Augsburg (SCHANA Study). Medicina. (2020) 56:280. 10.3390/medicina56060280 32517235 PMC7353873

[16] Huy C Schneider S. Instrument für die Erfassung der physischen Aktivität bei Personen im mittleren und höheren Erwachsenenalter: Entwicklung, Prüfung und Anwendung des “German-PAQ-50+”. Z Gerontol Geriatr. (2008) 41:208–16. 10.1007/s00391-007-0474-y 18327696

[17] Golicki D Niewada M Buczek J Karlinska A Kobayashi A Janssen MF. Validity of the Eq-5d-5l in stroke patients. Value Health. (2014) 17:A570. 10.1016/j.jval.2014.08.1906 27201901

[18] Li Kwah K Diong J. National Institutes of Health Stroke Scale (NIHSS). J Physiother. (2014) 60:61. 10.1016/j.jphys.2013.12.012 24856948

[19] Banks JL Marotta CA. Outcomes validity and reliability of the modified Rankin scale: implications for stroke clinical trials: a literature review and synthesis. Stroke. (2007) 38:1091–6. 10.1161/01.STR.0000258355.23810.c6 17272767

[20] Berger K Weltermann B Kolominsky-Rabas P Meves S Heuschmann P Böhner J. Untersuchung zur Reliabilität von Schlanganfallskalen. Die deutschen Versionen von NIHSS, ESS und Rankin Scale. Fortschr Neurol Psychiatr. (1999) 67:81–93. 10.1055/s-2007-993985 10093781

[21] Muruet W Rudd A Wolfe CDA Douiri A. Long-term survival after intravenous thrombolysis for ischemic stroke: a propensity score-matched cohort with up to 10-year follow-up. Stroke. (2018) 49:607–13. 10.1161/STROKEAHA.117.019889 29440582 PMC5839705

[22] McCarthy DJ Diaz A Sheinberg DL Snelling B Luther EM Chen SH. Long-term outcomes of mechanical thrombectomy for stroke: a meta-analysis. Sci World J. (2019) 2019:7403104. 10.1155/2019/7403104 31186620 PMC6521543

[23] Joundi RA Rebchuk AD Field TS Smith EE Goyal M Demchuk AM. Health-related quality of life among patients with acute ischemic stroke and large vessel occlusion in the ESCAPE trial. Stroke. (2021) 52:1636–42. 10.1161/str.52.suppl_1.P521 33691504

[24] Deb-Chatterji M Konnopka A Flottmann F Leischner H Fiehler J Gerloff C. Patient-reported, health-related, quality of life after stroke thrombectomy in clinical practice. Neurology. (2020) 95:e1724–32. 10.1212/WNL.0000000000010356 32680947

[25] Catto JWF Downing A Mason S Wright P Absolom K Bottomley S. Quality of life after bladder cancer: a cross-sectional survey of patient-reported outcomes. Eur Urol. (2021) 79:621–32. 10.1016/j.eururo.2021.01.032 33581875 PMC8082273

[26] Razdan SN Patel V Jewell S McCarthy CM. Quality of life among patients after bilateral prophylactic mastectomy: a systematic review of patient-reported outcomes. Qual Life Res. (2016) 25:1409–21. 10.1007/s11136-015-1181-6 26577764 PMC4867133

[27] Lo Buono V Corallo F Bramanti P Marino S. Coping strategies and health-related quality of life after stroke. J Health Psychol. (2017) 22:16–28. 10.1177/1359105315595117 26220458

[28] Huber MB Felix J Vogelmann M Leidl R. Health-related quality of life of the general German population in 2015: results from the EQ-5D-5L. Int J Environ Res Public Health. (2017) 14:426. 10.3390/ijerph14040426 28420153 PMC5409627

[29] Borchert K Jacob C Wetzel N Jänicke M Eggers E Sauer A. Application study of the EQ-5D-5L in oncology: linking self-reported quality of life of patients with advanced or metastatic colorectal cancer to clinical data from a German tumor registry. Health Econ Rev. (2020) 10:40. 10.1186/s13561-020-00297-6 33313984 PMC7733616

[30] Wit L Theuns P Dejaeger E Devos S Gantenbein AR Kerckhofs E. Long-term impact of stroke on patients' health-related quality of life. Disabil Rehabil. (2017) 39:1435–40. 10.1080/09638288.2016.1200676 27385479

[31] Grenthe Olsson B Sunnerhagen KS. Functional and cognitive capacity and health-related quality of life 2 years after day hospital rehabilitation for stroke: a prospective study. J Stroke Cerebrovasc Dis. (2007) 16:208–15. 10.1016/j.jstrokecerebrovasdis.2007.06.002 17845918

[32] Luengo-Fernandez R Gray AM Bull L Welch S Cuthbertson F Rothwell PM. Quality of life after TIA and stroke: ten-year results of the Oxford Vascular Study. Neurology. (2013) 81:1588–95. 10.1212/WNL.0b013e3182a9f45f 24107865 PMC3806919

[33] Xie J Wu EQ Zheng Z-J Croft JB Greenlund KJ Mensah GA. Impact of stroke on health-related quality of life in the noninstitutionalized population in the United States. Stroke. (2006) 37:2567–72. 10.1161/01.STR.0000240506.34616.10 16946158

[34] López pez F Portilla Cuenca JC Leno Díaz C Párraga Sánchez JM Gamez-Leyva G Casado Naranjo I. Sex differences in long-term quality of life after stroke: Influence of mood and functional status. Neurologia. (2020) 35:470–8. 10.1016/j.nrleng.2017.10.002 29273429

[35] Bigourdan A Munsch F Coupé P Guttmann CRG Sagnier S Renou P. Early fiber number ratio is a surrogate of corticospinal tract integrity and predicts motor recovery after stroke. Stroke. (2016) 47:1053–9. 10.1161/STROKEAHA.115.011576 26979863

[36] Carod-Artal FJ Egido JA. Quality of life after stroke: the importance of a good recovery. Cerebrovasc Dis. (2009) 27(Suppl. 1):204–14. 10.1159/000200461 19342853

[37] Oemrawsingh A van Leeuwen N Venema E Limburg M Leeuw FE Wijffels MP. Value-based healthcare in ischemic stroke care: case-mix adjustment models for clinical and patient-reported outcomes. BMC Med Res Methodol. (2019) 19:229. 10.1186/s12874-019-0864-z 31805876 PMC6896707

[38] Mukundan G Seidenwurm DJ. Economic and societal aspects of stroke management. Neuroimaging Clin North Am. (2018) 28:683–9. 10.1016/j.nic.2018.06.009 30322602

[39] Ghatnekar O Persson U Asplund K Glader E-L. Costs for stroke in Sweden 2009 and developments since (1997). Int J Technol Assess Health Care. (2014) 30:203–9. 10.1017/S0266462314000075 24893970

[40] Holmes MM Stanescu S Bishop FL. The use of measurement systems to support patient self-management of long-term conditions: an overview of opportunities and challenges. Patient Relat Outcome Meas. (2019) 10:385–94. 10.2147/PROM.S178488 31908555 PMC6924578

[41] Bruijn MAAM Synhaeve NE van Rijsbergen MWA Leeuw FE Mark RE Jansen BPW. Quality of life after young ischemic stroke of mild severity is mainly influenced by psychological factors. J Stroke Cerebrovasc Dis. (2015) 24:2183–8. 10.1016/j.jstrokecerebrovasdis.2015.04.040 26215135

[42] Rachpukdee S Howteerakul N Suwannapong N Tang-Aroonsin S. Quality of life of stroke survivors: a 3-month follow-up study. J Stroke Cerebrovasc Dis. (2013) 22:e70–8. 10.1016/j.jstrokecerebrovasdis.2012.05.005 22749628

[43] Arwert HJ Schults M Meesters JJL Wolterbeek R Boiten J Vliet Vlieland T. Return to work 2-5 years after stroke: a cross sectional study in a hospital-based population. J Occup Rehabil. (2017) 27:239–46. 10.1007/s10926-016-9651-4 27402347

[44] Bayerische Arbeitsgemeinschaft für Qualitätsicherung in der stationären Versorgung. Jahresauswertung Gesamt: Schlaganfall (2020). Available online at: URL: https://www.baq-bayern.de/media/file/1721.2019_851_BA_Gesamt.pdf (accessed January 2, 2020).

